# Iron Deficiency Generates Oxidative Stress and Activation of the SOS Response in *Caulobacter crescentus*

**DOI:** 10.3389/fmicb.2018.02014

**Published:** 2018-08-28

**Authors:** Laura Leaden, Larissa G. Silva, Rodolfo A. Ribeiro, Naara M. dos Santos, Alan P. R. Lorenzetti, Thiago G. P. Alegria, Mariane L. Schulz, Marisa H. G. Medeiros, Tie Koide, Marilis V. Marques

**Affiliations:** ^1^Departamento de Microbiologia, Instituto de Ciências Biomédicas, Universidade de São Paulo, São Paulo, Brazil; ^2^Departamento de Bioquímica e Imunologia, Faculdade de Medicina de Ribeirão Preto, Universidade de São Paulo, Ribeirão Preto, Brazil; ^3^Departamento de Genética e Biologia Evolutiva, Instituto de Biociências, Universidade de São Paulo, São Paulo, Brazil; ^4^Departamento de Bioquímica, Instituto de Química, Universidade de São Paulo, São Paulo, Brazil

**Keywords:** iron limitation, DNA damage, *Caulobacter crescentus*, oxidative stress, RNA-seq analysis

## Abstract

In *C. crescentus*, iron metabolism is mainly controlled by the transcription factor Fur (ferric uptake regulator). Iron-bound Fur represses genes related to iron uptake and can directly activate the expression of genes for iron-containing proteins. In this work, we used total RNA sequencing (RNA-seq) of wild type *C. crescentus* growing in minimal medium under iron limitation and a *fur* mutant strain to expand the known Fur regulon, and to identify novel iron-regulated genes. The RNA-seq of cultures treated with the iron chelator 2-2-dypiridyl (DP) allowed identifying 256 upregulated genes and 236 downregulated genes, being 176 and 204 newly identified, respectively. Sixteen transcription factors and seven sRNAs were upregulated in iron limitation, suggesting that the response to low iron triggers a complex regulatory network. Notably, *lexA* along with most of its target genes were upregulated, suggesting that DP treatment caused DNA damage, and the SOS DNA repair response was activated in a RecA-dependent manner, as confirmed by RT-qPCR. Fluorescence microscopy assays using an oxidation-sensitive dye showed that wild type cells in iron limitation and the *fur* mutant were under endogenous oxidative stress, and a direct measurement of cellular H_2_O_2_ showed that cells in iron-limited media present a higher amount of endogenous H_2_O_2_. A mutagenesis assay using the *rpoB* gene as a reporter showed that iron limitation led to an increase in the mutagenesis rate. These results showed that iron deficiency causes *C. crescentus* cells to suffer oxidative stress and to activate the SOS response, indicating an increase in DNA damage.

## Introduction

The metal iron is an essential micronutrient for bacterial growth. Under physiological conditions, it exists mainly in one of two redox states: the ferrous (Fe^2+^) form and the ferric (Fe^3+^) form. Iron is a prosthetic component for incorporation into proteins, and participates in important biological processes such as N_2_ fixation, tricarboxylic acid cycle, respiration, gene regulation, and DNA synthesis (Beinert et al., 1997; Andrews et al., 2003). Iron is not freely available in the environment since ferric iron is extremely insoluble, so bacteria have evolved strategies to ensure their physiological demands. Iron limitation leads to increased iron influx, the reduction in activity of non-essential iron enzymes, the activation of iron-independent pathways and the mobilization of protein-associated stored iron, as from ferritins. On the other hand, excess iron induces genes involved in its efflux and reduction of iron uptake to keep intracellular iron levels under strict regulation (Chandrangsu et al., 2017). This control, along with the activation of oxidative stress response, is an essential factor to allow the aerobic style of life (Touati, 2000).

*C. crescentus* is an aerobic free-living alphaproteobacterium that grows in oligotrophic aquatic environments, and despite apparently not synthesizing its own siderophores for iron uptake, is able to acquire them through TonB-dependent transporters (Balhesteros et al., 2017). In *C. crescentus*, four TonB-dependent transporters predicted to be involved in iron acquisition are regulated by Fur, and one of them was characterized as the hemin/hemoglobin transporter HutA (da Silva Neto et al., 2009; Balhesteros et al., 2017).

In most bacteria, the transcriptional regulator Fur tightly controls iron homeostasis (Fillat, 2014). Iron-bound Fur acts as transcriptional repressor under iron sufficient conditions, inhibiting the expression of genes for iron uptake and for non-essential iron enzymes (Andrews et al., 2003; Lee and Helmann, 2007). In several bacteria the repression by iron-Fur is released under iron limitation, allowing the expression of a small RNA (called RyhB in *Escherichia coli*, FsrA in *Bacillus subtilis,* PrrF1 and PrrF2 in *Pseudomonas aeruginosa*, NrrF in *Neisseria meningitidis,* and Bc_KC_sr1 and Bc_KC_sr2 in *Burkholderia cenocepacia* KC-01), which facilitates the degradation of the mRNA of non-essential iron enzymes (Masse and Gottesman, 2002; Wilderman et al., 2004; Masse et al., 2005, 2007; Mellin et al., 2007; Gaballa et al., 2008; Ghosh et al., 2017; Jackson et al., 2017).

Previously, a detailed *in silico* analysis of the promoter regions of the *nuoA-N* operon (NADH dehydrogenase), *acnA* (aconitase) and *sdhCBAD* (succinate dehydrogenase), confirmed by electrophoresis mobility shift assays, showed that Fur directly binds to these regulatory regions and activates the expression of these genes in response to iron (da Silva Neto et al., 2009), revealing that in *C. crescentus* the mechanism of iron control of metabolism is different than that of *E. coli*. Global transcriptional analyses using DNA microarrays allowed to define the Fur regulon, identifying 42 genes repressed and 27 genes activated by Fe-Fur (da Silva Neto et al., 2013).

In *C. crescentus*, Fur also activates the low oxygen signaling network FixK, FixT and FtrB cascade, suggesting that in *C. crescentus* iron regulation also can be sensitive to O_2_ levels (da Silva Neto et al., 2013). In *E. coli,* an anaerobic facultative bacterium, Fur activity is modulated by O_2_ levels (Beauchene et al., 2015). It has been reported that in anaerobic conditions the labile iron pool is higher than in aerobic conditions (Beauchene et al., 2017). Under anaerobic condition the upregulation of *feoABC* transport system facilitates the Fe^2+^ import increasing the labile iron pool, which is required to enhance the Fur regulon in these conditions (Beauchene et al., 2015, 2017).

The previous transcriptional analysis also showed that several genes have an iron-dependent and Fur-independent regulation in *C. crescentus,* such as those encoding the heat shock sigma factor RpoH and the Fe–S cluster biogenesis operon containing *iscR* (da Silva Neto et al., 2013). However, the tiling array platform used consisted of primers located mainly around the start codons of annotated protein-coding genes. As previous works using high-throughput RNA sequencing have identified an expressive number of non-coding RNAs that could participate in gene regulation (Landt et al., 2008; Schrader et al., 2014), it is important to identify those that participate in the regulatory network that control the response to iron levels.

In order to further understand the consequences of iron limitation, we carried out an RNA-seq-based transcriptomic analysis seeking to identify new genes responsive to iron limitation and belonging to the Fur regulon, as well as iron-regulated regulatory RNAs. Our analyses showed that under iron limitation several stress response genes are induced, and the cells are in a state of oxidative stress. Moreover, the results indicate that in *C. crescentus* under iron limitation, the genes involved in the SOS response are induced in a RecA-dependent manner, suggesting that in this condition the cells undergo DNA damage.

## Materials and Methods

### Bacterial Strains and Growth Conditions

The *Caulobacter crescentus* strains used were: NA1000 (wild-type) (Evinger and Agabian, 1977), SP0057 (*fur* mutant) (da Silva Neto et al., 2009), GM10 (*recA* mutant) (Galhardo et al., 2005), and NA1000 and GM10 harboring the pRKlacZ290 vector containing the *imuA* promoter (*imuA/lacZ* fusion) (Galhardo et al., 2005). Cultures were grown aerobically at 30°C in minimal medium (M2), which contains 10 μM FeSO_4_ (Ely, 1991). Iron-limiting conditions were obtained by adding the iron chelator 2,2-dipyridyl (DP) (Sigma, 100 μM) to the M2 medium (DP-treated M2) or by using a modified M2 medium without iron sulfate (iron-limited M2). Excess iron conditions were achieved by supplementing M2 medium with 100 μM FeSO_4_ instead of 10 μM FeSO_4_. The growth curves in each condition are shown in **Supplementary Figure S1**.

### RNA-seq Expression Profiling

The NA1000 and SP0057 strains (pre-cultivated in 3 ml M2 overnight) were diluted in duplicate into 10 ml M2 to an OD_600__nm_ of 0.1 and incubated at 30°C with agitation (250 rpm) until reaching OD_600__nm_ of 0.5. At this time DP was added to a final concentration of 100 μM to one aliquot of the NA1000 culture and these were further incubated for 2 h. Total RNA was isolated from independent cultures (1 ml) treated with RNAprotect Bacteria Reagent (Qiagen) and purified with Qiagen RNeasy Plus Mini Kit (Qiagen). RNA samples were quantified using NanoDrop 1000 (Thermo Scientific) and submitted to the removal of ribosomal RNA using Ribo-Zero Magnetic kit (Illumina). The quality of the isolated RNA and the percentage of rRNA present were checked with RNA 6000 Pico Kit using Agilent 2100 Bioanalyzer (Agilent). Complementary DNA was generated according to instructions of TruSeq RNA Sample Prep Kit (Illumina). The cDNA libraries were normalized to 4 nM using the Kapa Biosystems kit (Kapa Biosystems) for library quantitation prior to cluster generation. The samples were sequenced using the MiSeq (Illumina) platform according to the manufacturer’s instructions. RNA-seq experiments were carried out in biological duplicates.

cDNA reads were quality checked using Bioconductor’s 3.5 (Huber et al., 2015) Rqc 1.10.2 and trimmed using Trimmomatic 0.36 (Bolger et al., 2014) to remove adapters and low quality ends. Pre-processed reads were aligned to *C. crescentus* NA1000 reference genome using Bowtie 1.2 (Langmead et al., 2009) with the “-m 1” option to keep only uniquely mapped reads. Alignment files were further processed by SAMtools 1.3.1 (Li et al., 2009) and read counting was performed for each gene feature using GenomicAlignments 1.12.1 (Lawrence et al., 2013). The count matrix was input in DESeq2 1.16.1 (Love et al., 2014) and analyzed using group design. We generated two contrasts to find differentially expressed genes: (i) wt DP-treated for 2 h vs. wt and (ii) *fur* mutant vs. wt. Genes were considered differentially expressed if satisfying |log_2_fold change|≥ 1 and *q*-value < 0.05. We used information available in KEGG pathways (Kanehisa et al., 2017) to group differentially expressed genes in functional categories and also performed manual curation.

### Real-Time PCR

The gene expression profile was assessed by RT-qPCR. Cultures pre-cultivated in 3 ml M2 overnight were diluted into two aliquots of 10 ml M2 to an OD_600__nm_ of 0.05 and incubated at 30°C with agitation (250 rpm) until reaching OD_600__nm_ of 0.3. At this time DP was added to one aliquot to a final concentration of 100 μM and these were further incubated for 2 h. When iron limited M2 was used, the overnight culture was diluted in 10 ml M2, and when the cultures reached an OD_600__nm_ of 0.3 they were centrifuged for 20 min at 5,000 ×*g* and resuspended in the same volume of iron limited M2 (no iron added). One of the aliquots received FeSO_4_ to a final concentration of 10 μM, and the cultures were incubated at 30°C until the appropriate time. Total RNA was isolated from 5 ml cultures either in M2, iron limited M2 (with no iron addition) or DP-treated M2 for 2 h as indicated for each experiment. RNA was purified using TRIzol^®^ RNA (Invitrogen), treated with deoxyribonuclease I (Invitrogen) and complementary DNA was synthesized using the Superscript III First-Strand Synthesis System kit for RT-PCR (Invitrogen) with random hexamers. RT-qPCR reactions were performed on the Step One Plus Real Time PCR System (Applied Biosystems), using SYBR Green PCR Master Mix (Applied Biosystems) as detection reagent, in technical triplicates. RT-qPCR reactions were carried out in a final volume of 12 μl Power SYBR Green PCR master mix containing 10 μM of each primer, and 50 ng.μl^-1^ cDNA. The conditions were 95°C for 10 min, followed by 40 cycles comprising 95°C for 15 s and 60°C for 1 min. In order to confirm the amplification of a single product, dissociation curves were generated at the end of each PCR cycle. The 2^-ΔΔCt^ method was used to calculate the relative change in gene expression of each gene (Pfaffl, 2001), and the analysis was performed using the StepOne Software v.2.2. Specific primers were designed using primer3 software (Untergasser et al., 2012) for each gene for validation of RNA-seq data and gene CCNA_03876, coding the transcription termination factor Rho, used as reference control (**Supplementary Table S1**). All determinations were performed in biological duplicates and at least in technical triplicates and the mean values ± standard deviation (SD) are reported.

### β-Galactosidase Activity Assays

*C. crescentus* NA1000 or *ΔrecA* strains harboring the pRK*lacZ*290 vector containing the *imuA* promoter (*imuA/lacZ* fusion) were grown in M2 medium up to midlog phase (OD_600__nm_ of 0.2). Each culture of NA1000 was divided into four equal aliquots, and one was left without additions throughout the experiment. To two other aliquots, either 100 μM FeSO_4_ (called iron-added M2) or 100 μM DP (called DP-treated M2) were added. The fourth aliquot was centrifuged and the cells were suspended in M2 medium without iron (called iron-limited M2). In the assays with the *ΔrecA* strain, the culture was divided in two aliquots, one without additions and the second received 100 μM DP. The cultures were further incubated during the appropriate times and the β-galactosidase activity was determined (Miller, 1972).

### Microscopy

*C. crescentus* NA1000 and the *fur* mutant were grown in M2 medium up to midlog phase (OD_600__nm_ of 0.2–0.3). Each *C. crescentus* NA1000 culture was divided into equal aliquots, and one was left without additions throughout the experiment. To one other aliquot, DP to 100 μM was added and the cultures were further incubated for 2 h. One aliquot was centrifuged and the cells were suspended in the same volume of M2 medium without iron (iron-limited M2) for 4 h. To one of the aliquots H_2_O_2_ was added to 10 mM and incubated for 15 min. In the assays with the *fur* strain, the culture was divided in two aliquots, one was left without additions and the second received DP as above. Dihydrorhodamine 123 (Sigma D1054) was added to the cultures to a final concentration of 20 μM and the cultures were further incubated for 60 min. After the cells were washed they were resuspended in phosphate-buffered saline solution. The slides were observed using a fluorescein filter in a Nikon Eclipse TiE microscope, using a 100× objective and images were captured in an Andor EM CCD i-Xon camera.

### Survival and Mutagenesis Tests

For the mutagenesis tests, cultures of the *C. crescentus* NA1000 and *recA* strain were grown for 10 h in 10 ml of M2 medium. The cells from each culture were pelleted by centrifugation for 20 min at 5,000 ×*g* and resuspended in iron limited M2 (no iron added) to the same previous volume. The cultures were adjusted to OD_600nm_ of 0.2, and divided into two 5 ml aliquots. One of the aliquots received FeSO_4_ to a final concentration of 10 μM, and the cultures were incubated at 30°C with agitation for 12 h. One ml of each culture was centrifuged at 5,000 ×*g* and the cells were resuspended in 1 ml M2. Two hundred microliters were diluted in 1 ml M2 and incubated at 30°C with agitation for 24 h. Serial dilutions and plating on PYE were performed to determine the total number of viable cells. To determine the number of Rif-resistant mutants, 1 ml of each culture was plated on PYE medium containing 100 μg/ml rifampicin.

For the UV survival tests, *C. crescentus* NA1000, SP0057 and GM10 overnight cultures were diluted to an optical density at 600nm (OD_600nm_) of 0.05 in M2 medium. Each culture was divided into three equal aliquots, and to one (control) no additions were made throughout the experiment. One aliquot of the NA1000 culture was pre-incubated with 100 μM DP for 2 h. One aliquot was centrifuged and the cells were suspended in iron-limited M2 for the appropriate times. After the respective times, each culture was irradiated with 120 J/cm^2^ UV light (NA1000), 60 J/cm^2^ (SP0057) and 10 J/cm^2^ (GM10). Serial dilutions from each control and treated cultures were plated on M2 plates, and these were incubated in the dark for 48 h at 30°C for CFU counting.

### Analysis by HPLC-ESI/MS/MS of 8-oxo-dG

DNA extraction and enzymatic hydrolysis was carried out as described in Sanchez et al. (2018) with the following modifications. The bacterial pellet was resuspended in lysis buffer containing 0.1 mM desferroxamine. Treatment with 60 μL of proteinase K (20 g/L) was performed together with 675 μL of a 10% (w/v) solution of SDS for 2 h at 37°C. The DNA pellet was solubilized in a 3 mM solution of desferroxamine mesylate, and concentration was measured by A_260__nm_. For the hydrolysis of 30 μg of DNA, 5 μl of 3 M sodium acetate buffer pH 5.0 and 4 U of nuclease P1 were added to the sample, which was incubated at 37°C for 30 min at 450 rpm. Following this step, 5 μl of 3 M Tris-Cl buffer pH 7.4 and 5 μl of phosphatase buffer containing 100 mM Tris-Cl, 5 mM MgCl_2_, 0.2 mM ZnCl_2_ and 50% (v/v) glycerol were added. After addition of 2 U of alkaline phosphatase, the reaction mixture was incubated at 37°C for 1 h at 450 rpm.

Online HPLC/ESI/MS-MS analyses were carried out as described in Garcia et al. (2010) using an API-4000 QTRAP mass spectrometer (Applied Biosystems, Foster City, CA, United States). 8-oxo-dG in the DNA samples were detected by selected reaction monitoring (SRM) using an analytical column (Luna C18(2), 250 mm × 4.6 mm i.d., 5 μm, Phenomenex, Torrance, CA, United States). 8-oxo-dG was eluted from this column with a gradient of water and acetonitrile containing 0.1% formic acid with the following method: from 0 to 10 min, 5 to 12% acetonitrile, and 0.5 to 0.25 mL/min; from 10 to 15 min, 12% acetonitrile and 0.25 mL/min; from 15 to 40 min, 12 to 40% acetonitrile and 0.25 mL/min; from 40 to 41 min, 40 to 95% acetonitrile and 0.25 to 0.5 mL/min; from 41 to 45 min, 95% acetonitrile and 0.5 mL/min; from 45 to 46 min, 95 to 5% acetonitrile and 0.5 mL/min; from 46 to 50 min, 5% acetonitrile and 0.5 mL/min. An isocratic pump was used to simultaneously load a second column [Luna C18(2), 150 mm × 2 mm i.d., 3 μm, Phenomenex, Torrance, CA, United States]. The DNA hydrolysates containing 3.6 fmol of the [15N5]-8-oxo-dG internal standards were injected into the system described above. The [M + H]+ ions corresponding to the m/z values 284.1/167.9 (8-oxo-dG) and 289.1/172.9 ([15N5]-8-oxo-dG), were monitored with a dwell time of 150 ms.

### H_2_O_2_ Dosage Assays

Colonies from the NA1000 strain were inoculated in 3 ml of M2 medium and grown for 16 h at 30°C with agitation. These two independent cultures (biological replicas) were then diluted to an OD_600nm_ of 0.1 in 15 ml of M2 and incubated at 30°C with agitation until reaching an OD of 0.25. The cultures were divided into four 7 ml aliquots, and cells were pelleted by centrifugation for 20 min at 5,000 rpm. Cells from two aliquots were suspended in M2 and from the other two in iron limited M2 (no iron added) in the same previous volume. One culture in M2 and one culture in iron limited M2 received DP to a final concentration of 100 μM for each replica. The cultures were incubated for 3 h at 30°C with agitation. For the assays 1.5 ml from each culture was centrifuged at 12,000 ×*g* and the cells resuspended in 100 μl of 20 mM Tris-Cl pH 7.4.

Determination of the amount of H_2_O_2_ was carried out as described in Barros et al. (2003). Briefly, 50 μl of each bacterial suspensions were added to 3 ml of a solution containing 50 μM Amplex Red (Thermo Scientific) and 1.0 U/ml horseradish peroxidase (HRP; Sigma-Aldrich), in 20 mM Tris-Cl pH 7.4. The rate of Amplex Red oxidation to the fluorescent product (resorufin) was analyzed at 25°C using a Cary Eclipse (Varian) fluorescence spectrophotometer equipped with continuous stirring.

### Statistical Analyses

Error bars shown on graphs indicate standard deviations or standard error, as indicated. Statistical significance was tested by one-way ANOVA using the Tukey post-test for multiple comparisons or by *t*-test when only two conditions were being compared.

## Results

### Expanding the Low Iron Stimulon in *C. crescentus*

**FIGURE 1:**
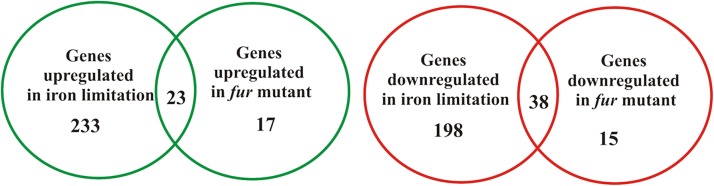
Overview of the *C. crescentus* iron-regulated and Fur-regulated genes identified by RNA-seq analyses. The Venn diagrams indicate the number of up-regulated (green) and down-regulated (red) genes from experiments comparing wild type cells exposed to DP-treated M2 versus M2 or comparing the *fur* mutant strain *versus* wild type strain both in M2. Differentially expressed genes were considered those with log_2_ fold change ≥1 or ≤1 and *q*-value < 0.05.

Previous works using DNA microarrays and bioinformatics have characterized the iron limitation response in *C. crescentus* in the rich amino acid-based medium PYE (da Silva Neto et al., 2009, 2013). In that case, PYE was supplemented with 100 μM FeSO_4_ or 100 μM DP, respectively, to allow identification of differentially expressed genes. In order to further characterize the response to iron limitation of this oligotrophic bacterium, RNA-seq-based global transcriptional analysis was performed from cells grown in M2 minimal medium. The RNA-seq experiments were carried out using RNA samples prepared from two independent biological cultures. To define the *C. crescentus* iron-regulated genes we compared the transcriptome of wt cells grown in M2 treated or not with the iron chelator 2′,2-dypiridyl for 2 h (DP-treated). The addition of DP significantly altered the expression of 492 genes (256 upregulated genes and 236 downregulated genes) (**Figure 1**). To identify the genes regulated by Fur, we compared the transcriptome of wt cells with the *fur* mutant in M2. Overall, 93 genes were differentially expressed in the *fur* mutant (40 upregulated genes and 53 downregulated genes) (**Figure 1**).

We found 23 genes upregulated and 38 genes downregulated both in DP-treated condition and in the *fur* mutant (**Supplementary Table S2**). Seventy-four percent (17 genes) of the upregulated genes were genes previously identified by DNA microarray analysis in PYE, and six genes are new. Of these, four genes encoded hypothetical proteins, one a serine protease (CCNA_01341) and another the amelogenin/CpxP-related protein (CCNA_03997). Of the 38 downregulated genes, 11 genes were previously known as iron- and Fur-regulated in PYE, and 17 genes belong to the regulon controlled by FixL-FixK-FixT, among them the *cytbb3* operon (CCNA_01467-68-69-70-71-72-73), CCNA_01475 encoding the family outer membrane cation channel (*ompW*) and CCNA_01476-77 that encode the CRP-family transcription regulator (*ftrB*) and oxygen-independent coproporphyrinogen-III oxidase (*hemN*). In agreement with the results obtained with DNA microarrays, these data indicate that Fur might activate, probably indirectly, the oxygen signaling network.

In general, the Fur regulon determined in M2 (23 genes) was smaller than that previously determined in PYE (42 genes), while the number of genes induced only in DP-treated condition was larger in M2 (233 genes versus 66 genes upregulated in PYE). Interestingly, some genes that are regulated by Fur and were highly responsive to DP-treatment in PYE (da Silva Neto et al., 2009, 2013) did not show an increase in their transcript levels in DP-treated M2 medium. For example, CCNA_03023 that encodes a TonB-dependent receptor and CCNA_02275 that were upregulated gene in DP-treated PYE (15.3- and 64.8-fold, respectively) were below the cutoff in our experimental conditions. Still, these genes were upregulated in the *fur* mutant (CCNA_03023, 3.3-fold and CCNA_02275 5-fold), confirming that their expression is under Fur regulation, although the levels of expression are differently modulated according to the culture medium (**Supplementary Table S2**).

Eight previously predicted sRNAs (Landt et al., 2008; Schrader et al., 2014) were identified as differentially expressed in our experiments. One of these (R0117) was upregulated both in DP-treated condition (16.3-fold) and in the *fur* mutant (3.6-fold). This gene encodes a 1-kb RNA that maps to a region where previously annotated transcripts were shown to be regulated by Fur (da Silva Neto et al., 2013). One sRNA gene (R0088) was upregulated only in the *fur* mutant (7.7-fold), while six (R0180, R0119, R0116, R0049, R0080, and R0077) were upregulated only in DP-treated condition, varying from 3.6-fold for R0077 to 45.7-fold for R0180 (**Supplementary Table S2**). We could not identify a conserved Fur-box upstream of any of the sRNAs, including R0088, suggesting that it is indirectly regulated by Fur. The other six sRNAs could be important for the iron limitation response, in a Fur-independent manner, possibly regulating the expression of subsets of genes.

### Several Transcription Factors Are Induced in Iron Limitation

**FIGURE 2:**
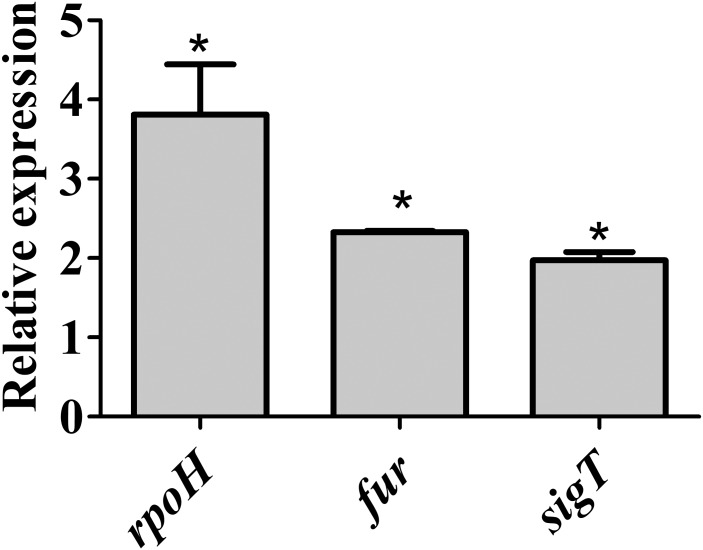
Expression of *fur*, *sigT*, and *rpoH* in wild type strain under iron limitation. Gene expression was determined by RT-qPCR using RNA from cells grown in iron limited M2 (for 4 h) relative to the expression in M2. Bars with asterisks (^∗^) are significantly different to wt growing in M2 medium (*P* < 0.05) by Student’s *t*-test.

In M2 treated with DP the induction of 16 genes encoding transcription regulators was observed (**Supplementary Table S2**), of which several are involved in controlling stress response in *C. crescentus*. These include the RNA polymerase sigma factor *rpoH* (8.5-fold) and three ECF sigma factors (σ^E^, σ^T^, and σ^U^, 5.9-, 5.5-, and 12.7-fold, respectively) as well as the σ^T^ regulatory systems LovR/LovK (*lovR*, 5.3-fold) and PhyR/PhyK/NepR (3.9-, 4.8-, and 6.7-fold, respectively) (Foreman et al., 2012). While the RpoH and several heat shock genes were previously identified as induced in DP-treated PYE (da Silva Neto et al., 2013), the other systems were identified only in the conditions used in this work. Surprisingly, the *lexA* gene encoding the SOS system regulator was also induced in DP-treated condition (8.4-fold), along with many genes belonging to the LexA regulon that will be discussed below (**Supplementary Table S2**). *fur* was not classified as upregulated in RNA-seq analysis although it presented a twofold induction, because it did not attend our statistical criteria. To further verify the expression of these regulators under iron limitation, we used RT-qPCR to measure the transcript levels of *fur, rpoH,* and *sigT*. The results show that all genes were induced in iron limitation generated by resuspending the cells in M2 without added iron (**Figure 2**).

### Iron Deficiency Induces the SOS Response

**FIGURE 3:**
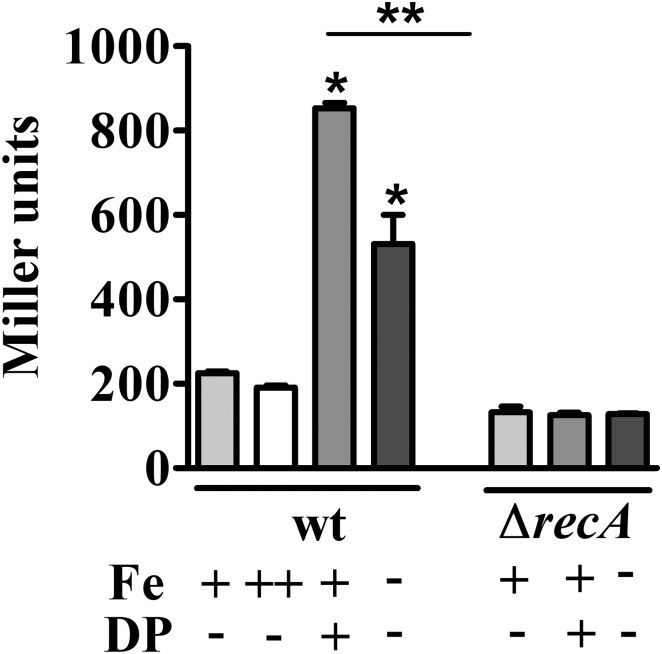
Determination of iron-dependent regulation of *imuA* expression. Expression was determined by β-galactosidase activity assays of each strain harboring *imuA/lacZ* transcriptional fusions. Cultures were grown in either M2 (Fe+/DP–), iron-supplemented M2 (with 100 μM FeSO_4_, Fe++/DP–), DP-treated M2 for 4 h (with 100 μM DP, Fe+/DP+) or iron-limited M2 (Fe–/DP–) for 4 h. The *ΔrecA* mutant was grown in the same conditions, except for iron supplemented M2. Bars with asterisks (^∗^) are significantly different to wt grown in M2; and (^∗∗^) indicates that results from DP-treated M2 and iron-limited M2 are significantly different (*P* < 0.05) by one-way ANOVA test.

To discriminate whether the upregulation of genes belonging to the SOS response was caused by iron limitation or due to a secondary effect of the iron chelator used, the expression of the *imuA-imuB-dnaE2* operon was measured by β-galactosidase activity using a transcriptional fusion to *lacZ* (P*imuA-lacZ*) (**Figure 3**). This operon is induced in response to DNA lesions in a RecA-dependent manner, and the three genes are required for the error-prone processing of DNA lesions (Galhardo et al., 2005). The results showed that *imuA* is induced under iron limitation conditions in both DP-treated M2 (containing the chelator DP) and in iron-limited M2 (no iron added), compared to M2 or iron-supplemented M2. The levels of β-galactosidase activity were slightly different between DP-treated M2 and iron-limited M2, but both were higher than the controls. These results indicated that the SOS response is being activated by iron limitation, suggesting that DNA damage is occurring in this condition. In order to verify if *imuA* induction was in fact responding to the SOS regulators, we analyzed *imuA* expression in the *recA* mutant (**Figure 3**). The results showed that the induction of *imuA* under iron limitation is dependent on RecA, indicating that it was induced by the SOS regulatory system.

### Iron Deficiency Generates Oxidative Stress

The genes encoding the catalase-peroxidase and two superoxide dismutases, as well as several genes coding for oxidative stress response (glutaredoxin, glutathione *S*-transferase, peroxiredoxin, glutathione synthetase, thioredoxin) were upregulated upon DP-treated conditions (**Supplementary Table S2**). To determine if the reason for the activation of SOS response could be a state of oxidative stress, we tested if iron limitation could cause oxidative stress in *C. crescentus*.

**FIGURE 4:**
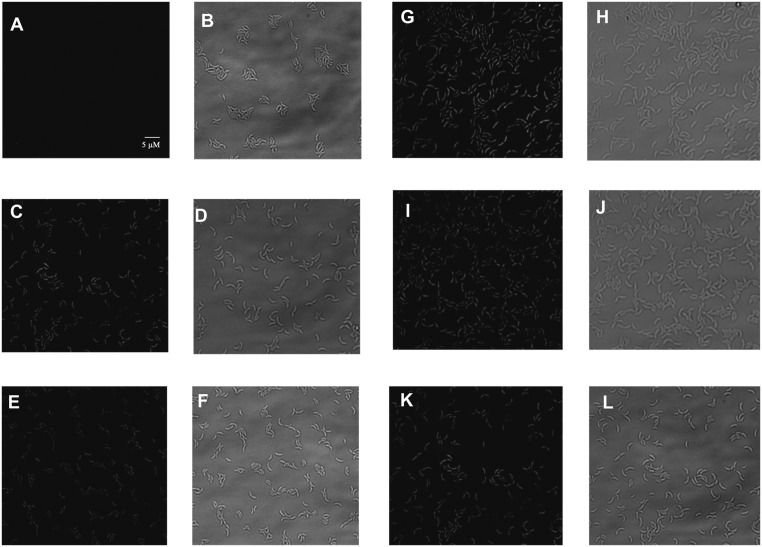
Determination of the oxidative stress state of *C. crescentus* NA1000 in response to iron levels. Wild type NA1000 strain cultures were grown in M2 **(A,B)**, DP-treated M2 (DP for 2 h) **(C,D)**, or iron-limited M2 for 4 h **(E,F)**; and cultures of the *fur* mutant were grown in M2 **(G,H)** or DP-treated M2 for 2 h **(I,J)**. As a control, NA1000 cultures received H_2_O_2_ for 5 mM and incubated for 15 min **(K,L)**. Samples were treated with dihydrorhodamine 123 and analyzed by fluorescence microscopy using a fluorescein filter **(A,C,E,G,I,K)** and under light microscopy **(B,D,F,H,J,L)**.

Firstly, this was done by incubating exponential phase cells with dihydrorhodamine 123. This compound is able to penetrate the cells and becomes fluorescent as a result of intracellular oxidation. The cells were analyzed by fluorescence microscopy (**Figures 4A,C,E,G,I,K**) and light microscopy (**Figures 4B,D,F,H,J,L**) to verify the amount of cells with visible fluorescence. While the wt cells in M2 showed no fluorescence (**Figures 4A,B**), when exposed to 100 μM DP (**Figures 4C,D**) or grown in iron-limited medium (**Figures 4E,F**) all cells were fluorescent after 2 h, confirming that iron limitation leads to an oxidative state. Interestingly, the *fur* mutant showed fluorescence both in M2 medium (**Figures 4G,H**) and in the presence of 100 μM DP (**Figures 4I,J**), suggesting that Fur could be involved in preventing oxidative stress in *C. crescentus*. As a positive control, wt cells were incubated with H_2_O_2_ for 15 min (**Figures 4K,L**).

As a second confirmation of these results, determination of H_2_O_2_ in NA1000 cultures was carried out by measuring the rate of oxidation of Amplex Red in the presence of HRP. Cells from NA1000 cultures grown either in M2, or incubated for 3 h in iron limited M2 (no iron added), DP-treated M2 or DP-treated iron limited M2 were used for H_2_O_2_ determination (**Table 1**). The results showed that in the three cultures subject to iron limitation the rate of Amplex Red oxidation was 2- to 2.5-fold higher than in M2 (*P* < 0.05), indicating that there is more endogenous H_2_O_2_, which could be measured in intact cells since it is freely diffusible.

**Table 1:** Determination of hydrogen peroxide generated in *C. crescentus* NA1000 strain in different growth conditions.

Growth condition	pmol H_2_O_2_/DO/min^c^
M2	20.81 ± 2.43
Iron limited M2^a,d^	51.50 ± 3.17
DP-treated M2^b,d^	49.50 ± 4.30
DP-treated iron limited M2^a,b,d^	42.91 ± 1.67


^*a*^The cells pellets were resuspended in iron limited M2 (no iron added) in the same previous volume. See “Materials and Methods” section for details. ^*b*^The culture was incubated with 100 μM DP for 3 h. ^*c*^The results are the average of two independent assays ±SD. ^*d*^Significantly different from the M2 growth condition (*P* < 0.05) by one-way ANOVA test.

**FIGURE 5:**
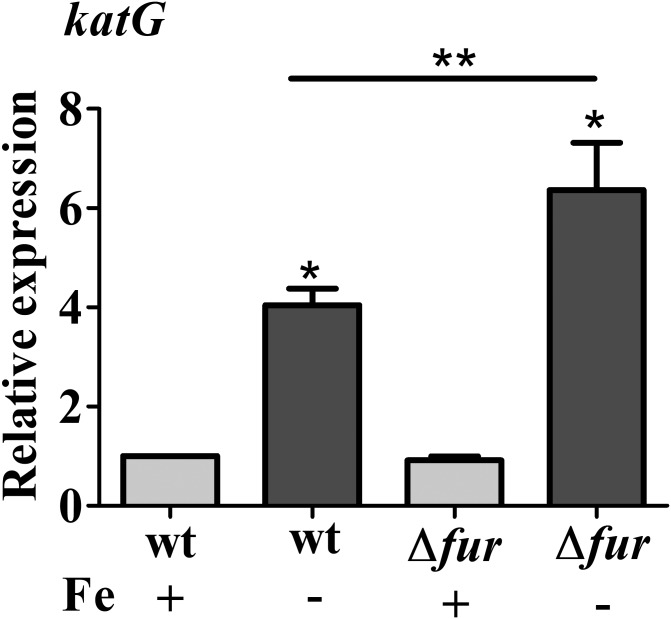
Expression of *katG* in response to iron limitation. Expression was determined by RT-qPCR from NA1000 (wt) and *fur* mutant (*Δfur*) cultures grown in M2 or iron limited M2 (no iron added) for 4 h. Bars with asterisks (^∗^) are significantly different from wt cells in M2 medium (^∗∗^) indicates that the results of *Δfur* and *Δfur* in iron limited M2 are significantly different from each other (*P* < 0.05 by one-way ANOVA test).

Previously, it was shown that OxyR activates the expression the *C. crescentus katG* gene in response to hydrogen peroxide, and *katG* was not regulated by Fur (Italiani et al., 2011). To determine whether the oxidative stress observed in the *fur* mutant and in the wt cells under iron limitation could lead to the OxyR-mediated response, the expression of *katG* was measured by RT-qPCR (**Figure 5**). However, there is still an increase in *katG* expression in the *fur* mutant in iron limited M2 as compared to M2, to a similar extent as in wt, suggesting that iron limitation is causing this induction.

Interestingly, several toxin-antitoxin encoding genes were highly upregulated in DP-treated cells, such as the ParD2/ParE2 (22.8- and 23.3-fold induction, respectively), the antitoxin protein RelB3 (23.6-fold), the HigB toxin (17.2-fold) and the DNA replication inhibitor toxin SocB/antitoxin SocA (17.2- and 14.0-fold induction, respectively). Previous work has shown that ψ-*parDE2* and *relBE3* are induced in response to oxidative stress (Fiebig et al., 2010). The *higB* gene is repressed by LexA, and HigB plays a role in mediating the intensity of the response to antibiotics together with the SOS response, in a regulatory interplay between LexA, HigBA, and AcrAB2-NodT (Kirkpatrick et al., 2016). In fact, in the DP-treated cells the *acrAB2* genes were also induced threefold to fivefold.

### The SOS Response Is Induced Under Iron Deficiency

**FIGURE 6:**
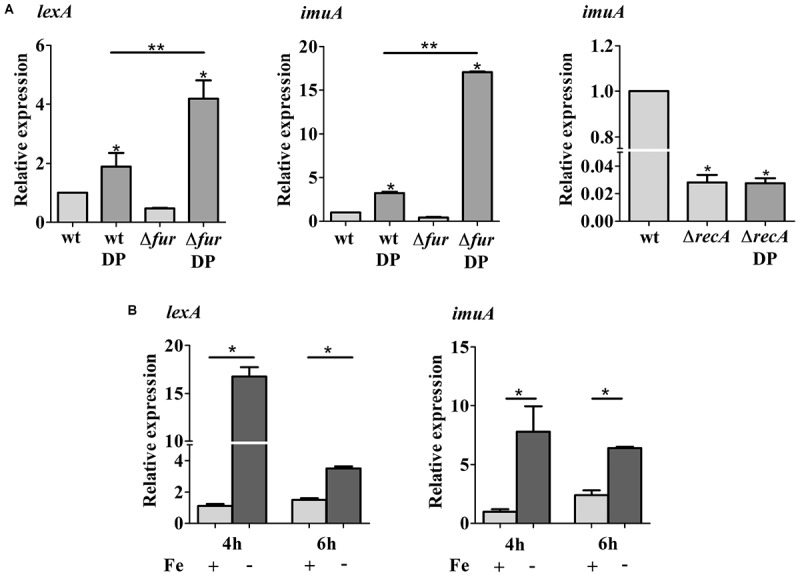
Expression of *lexA* and *imuA*. **(A)** Gene expression was assessed by RT-qPCR from wild type NA1000 (wt), the Fur mutant (*Δfur*) and *recA* mutant (*ΔrecA*) cultures grown in M2 and DP-treated M2 (DP) for 2 h. **(B)** Gene expression was assessed by RT-qPCR from NA1000 cultures grown in M2 and iron limited M2 (–Fe) for 4 h and 6 h. Bars with asterisks (^∗^) are significantly different from wt in M2 medium; (^∗∗^) indicates that the results. Gene expression was assessed by RT-qPCR from wild type NA1000 (wt), the Fur mutant (*Δfur*) and *recA* mutant (*ΔrecA*) cultures grown in M2 and DP-treated M2 (DP) are significantly different from each other (*P* < 0.05) by one-way ANOVA test.

The activation of SOS response in DP-treated *C. crescentus* cultures suggests that DNA damage is being generated in this condition. To establish the role of Fur in the SOS signaling network, the transcripts levels of *lexA* and *imuA* were measured by RT-qPCR in wt and the *fur* mutant cells grown in either M2 medium or DP-treated M2 for 2 h (**Figure 6A**). In DP-treated NA1000 cultures, both transcripts were upregulated twofold (*lexA*) and fourfold (*imuA*) in wt cells, whereas in the *fur* mutant the expression was increased 3- and 16-fold comparing to wt in M2 medium, respectively. This induction is not observed in the *recA* mutant (**Figure 6A**). Since the *fur* mutant in M2 is under oxidative stress, but does not show an upregulation of the SOS response, this suggests that iron deficiency, and not the oxidative stress generated, is triggering the activation of SOS response. In fact, when both were grown in DP-treated medium, the SOS response is highly induced. The results also indicate that Fur may have a protective role against the resulting DNA damage, since the induction of both genes in the *fur* mutant was much higher than in the wt. To confirm that this effect was due to the iron limitation, the assays were repeated with the NA1000 strain grown in M2 or iron limited M2 (no iron added) (**Figure 6B**). The results were the same as with the DP-treated cultures, confirming that iron limitation is causing this response.

In order to directly measure the increase in the levels of DNA damage that could be occurring in response to iron deprivation, we measured the amount of 8-oxo-dG in the DNA of cells incubated in M2 or iron limited M2 (no iron added) for 12 h. The results did not show any significant difference between the two treatments (M2, 8.45 ± 5.4 8-oxo-dG/10^5^ dG, and M2 with no iron 8.18 ± 2.71 8-oxo-dG/10^5^ dG), although the levels of 8-oxo-dG detected were at the limit of detection. However, we have to consider that this type of base alteration may not be the most indicative for *Caulobacter*, and other types of oxidative lesions may be occurring.

**FIGURE 7:**
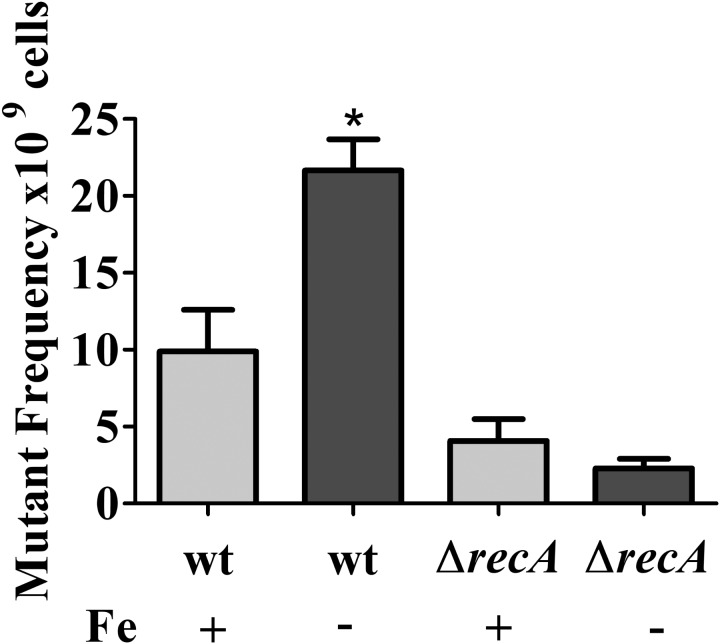
Effect of iron limitation on mutagenesis. Midlog phase cultures of *C. crescentus* NA1000 were grown in M2 (Fe+) or iron limited M2 (Fe–) (see the section “Materials and Methods”), and the cultures were incubated for 12 h. Cultures were plated on PYE to determine the total CFU counts. To determine the number of Rif-resistant mutants, 1 ml of each culture after treatment was plated on PYE with 100 μg/ml rifampicin. The relative mutant frequency was calculated by dividing the average of the Rif-resistant CFU by the average of the total CFU. The experiments were done in at least six independent biological assays with two technical replicas each. Bars with asterisks (^∗^) are significantly different to the wt control (*P* < 0.05) by one-way ANOVA test. Bars indicate standard error.

In the mutagenesis tests, cultures of NA1000 and *recA* strains grown in M2 or iron limited M2 for 12 h were plated on PYE containing 100 μg/ml rifampicin. The results showed that the NA1000 cultures grown in iron limited medium presented an increase in the amount of Rif-resistant mutants (**Figure 7**), but this is not the case for the *recA* mutant. These results indicate that iron deprivation leads to an increase in mutagenesis in *C. crescentus*, mediated by the SOS response. These results were consistent with the upregulation of the SOS genes in the same conditions, suggesting that increase in mutagenesis might be a result of error-prone DNA polymerases activity.

**FIGURE 8:**
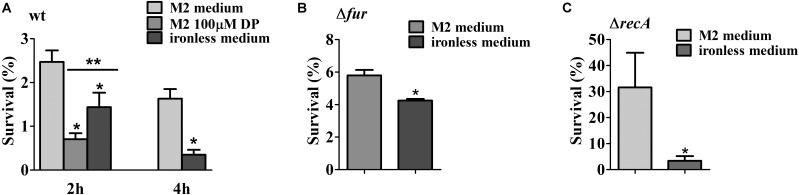
Survival test of the NA1000 strain exposed to UV light. **(A)** NA1000 cultures were grown either in M2, DP-treated M2 for 2 h or iron-limited M2 for 2 h or 4 h and then irradiated with 120 J/cm^2^ UV-light. **(B)** SP0057 cultures were grown either in M2, or iron-limited M2 for 4 h and then irradiated with 60 J/cm^2^ UV-light. **(C)** GM10 cultures were grown either in M2, or iron-limited M2 for 4 h and then irradiated with 10 J/cm^2^ UV-light. Survival was determined relative to the colony counts at time 0 h, before irradiation (100%). The results shown are the average of three independent experiments. Bars with asterisks (^∗^) are significantly different to M2 medium; (^∗∗^) indicates that DP-treated M2 and iron-limited M2 are significantly different (*P* < 0.05) by one-way ANOVA test **(A)** or Student’s *t*-test **(B,C)**.

The activation of DNA repair enzymes protects the DNA against exogenous physical agents like UV light. In order to verify if cells under iron limitation were more resistant to UV light-induced damage due to the activation of the SOS response, midlog phase cultures of the NA1000 strain grown in M2 were divided into aliquots. One was left without addition, one received 100 μM DP for 2 h, and in two samples cells were centrifuged and resuspended in iron-limited M2 and further incubated for either 2 h or 4 h (**Figure 8A**). The cultures were then irradiated with 120 J/cm^2^ UV light, and the results showed that cells that were in iron limitation were more sensitive to UV than those without treatment. The same result was obtained with the *fur* mutant, (irradiated with 60 J/cm^2^ UV light), and the *recA* mutant (irradiated with 10 J/cm^2^ UV light) grown in M2 or iron limited M2 (**Figures 8B,C**). This indicates that the previous activation of the SOS response in iron limitation is not only not sufficient to mount a protection against damage caused by UV light, but the cells being already stressed contributes for a poor response to UV-generated stress.

## Discussion

The Fur regulon in *C. crescentus* grown in rich amino acid-based medium was extensively studied and its targets were determined in previous works (da Silva Neto et al., 2009, 2013). However, in minimal medium cells are in a metabolic biosynthetic mode, more similar to the oligotrophic environments where this bacterium thrives, so the consequences of iron limitation for *C. crescentus* grown in these conditions were investigated. The previous whole transcriptome study was carried out in PYE medium that received either 100 μM FeSO_4_ or of 100 μM DP, which allowed the Fur regulon to be almost completely identified. In this work, the M2 medium was compared with DP-treated M2 medium, and allowed a complementary identification of iron limitation-induced genes. As expected, in defined M2 medium the genes for nitrogen and amino acid metabolism that were differentially expressed in DP-treated PYE were not identified, showing that the growth conditions affect the iron limitation response.

Among the iron limitation-induced genes were those encoding seven predicted small RNAs (sRNAs) (Landt et al., 2008; Schrader et al., 2014), being only one differentially expressed in the *fur* mutant compared to wt. The regulatory networks of several bacteria show that Fur is directly regulating the expression of iron-regulated regulatory RNAs (Masse and Gottesman, 2002; Wilderman et al., 2004; Masse et al., 2005, 2007; Mellin et al., 2007; Gaballa et al., 2008; Ghosh et al., 2017; Jackson et al., 2017). Although in this work we could detect only one Fur-regulated sRNA, we cannot exclude the possibility that there are some sRNAs that are regulated by Fur in other medium conditions. One caveat of the method used is that the RNA-seq was not strand-specific, therefore we may not detect all the differentially expressed sRNAs genes when they overlap with other genes. A more thorough investigation will bring light to this matter.

**FIGURE 9:**
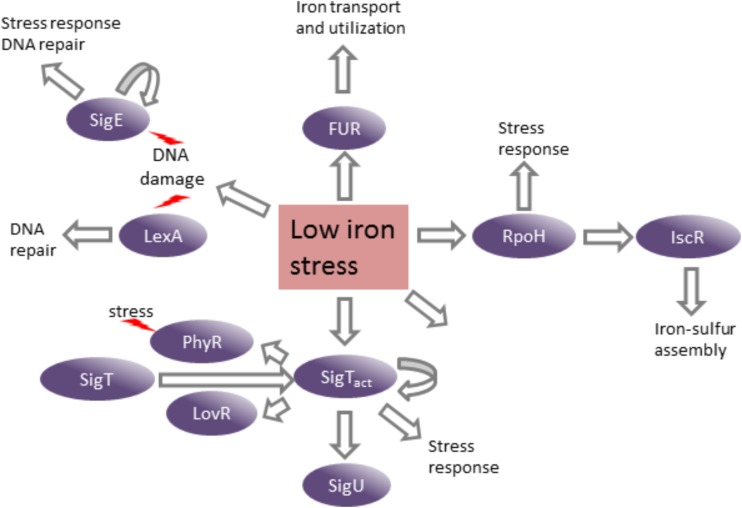
Consequences of low iron stress on *C. crescentus* gene expression. The induction of several transcription factors in response to stress generated by iron limitation leads to the activation of their regulons and distinct stress responses. Curved arrows indicate autoregulation.

The genes encoding several transcription factors were upregulated in the DP-treated medium in a Fur-independent manner, which could be mediating the regulation of subsets of genes in response to the stress generated by iron limitation (**Figure 9**). Three extracytoplasmic function (ECF) sigma factors (σ^T^, σ^U^, and σ^E^) regulate genes involved in protecting cells against stress, mainly oxidative damage. It has been shown that the σ^E^ is responsive to singlet oxygen, UV-A, cadmium, and organic hydroperoxide (Lourenço and Gomes, 2009). The SigT regulon is important for the response to osmotic and oxidative stresses (Alvarez-Martinez et al., 2007; Britos et al., 2011; Lourenço et al., 2011). Although *sigT* and most of its known regulon fell below our cutoff criterion in the *fur* mutant, *sigT* and *sigU* were highly activated in the DP-treated medium.

The induction of several genes related to oxidative stress response was in agreement with an endogenous oxidative stress state identified in *C. crescentus* cells under iron limitation. Since *katG*, the genes for superoxide dismutases, and several redoxins were upregulated in iron limitation, this indicates that the oxidative stress observed is caused by reactive oxygen species (ROS) of different types. The increase of oxidative stress under iron limitation has also been observed in the cyanobacterium *Anabaena* sp., but not in *E. coli* or *B. subtilis*. Iron starved *Anabaena* PCC7120 exhibit 100-fold increase of ROS compared to non-starved cells (Latifi et al., 2005, 2009). Interestingly, a striking difference is that *Caulobacter* and *Anabaena* are strict aerobes, while *E. coli* and *B. subtilis* can grow in anaerobiosis. In *Anabaena* PCC7120, the FurA regulator has been shown to control genes related to protection against oxidative stress (Gonzalez et al., 2016). Contrasting with this, *E. coli* does not exhibit a significant increase in the ROS levels under iron limitation (Latifi et al., 2005), and it has been proposed that Fur represses iron uptake as an adaptive response to protect against hydroxyl radicals generated by Fenton chemistry (Faulkner and Helmann, 2011). In *B. subtilis* the upregulation of genes encoding enzymes for peroxide stress response (*ahpCF, mrgA,* and *katA*) was observed in iron limitation (Baichoo et al., 2002); however, there was no increase of ROS levels in cells in these conditions (Latifi et al., 2005). The activation of these genes in *B. subtilis* can be explained by the fact that *fur* belongs to the PerR regulon (Baichoo et al., 2002; Faulkner and Helmann, 2011). Under iron deficiency PerR becomes an inactive repressor leading to the activation of *fur*, *ahpCF, mrgA,* and *katA* gene expression (Faulkner and Helmann, 2011).

Interestingly, the gene encoding the ferritin-like DNA-binding protein Dps was induced specifically in DP-treated conditions. In *E. coli* Dps confers protection against oxidative stress caused by H_2_O_2_, and it was proposed that this is due its ability to bind DNA and inhibit the Fenton reaction. In *C. crescentus*, *dps* is regulated by SigT (Lourenço et al., 2011) and Dps has an important role in protecting the cell from oxidative stress (de Castro Ferreira et al., 2016). However, while in *E. coli* *dps* is induced by starvation and regulated by OxyR under oxidative stress (Almiron et al., 1992; Altuvia et al., 1994), in *C. crescentus* its regulation is not responsive to hydrogen peroxide, and is independent of Fur and OxyR (de Castro Ferreira et al., 2016). Its regulatory pattern indicates that its main role in *C. crescentus* is protecting the DNA against the damage generated in low iron condition.

The most remarkable difference in the stimulon determined in this work was the induction of the SOS regulon. The presence of single stranded DNA activates the recombinase RecA that stimulates the autocatalytic cleavage of the transcriptional repressor LexA, inducing the SOS regulon that is comprised of DNA repair genes. The SOS regulon in *C. crescentus* was previously characterized (da Rocha et al., 2008; Modell et al., 2011). Twenty-two genes involved in DNA repair (18 belonging to SOS regulon) were upregulated in DP-treated medium, including *lexA* and *recA*. We showed that the iron limitation induction of *imuA,* as an SOS indicator gene, was dependent on RecA, so the induction of the SOS regulon was caused by the presence of DNA damage. Moreover, two genes (CCNA_01596 and CCNA_02930) that are induced by genotoxic treatments even though they do not belong to the SOS regulon are also upregulated in response to DP treatment (da Rocha et al., 2008; Modell et al., 2011, 2014), suggesting the response to DNA damage is broader than just the SOS system. Unexpectedly, despite the increase in expression of some genes belonging to the SOS regulon, cells grown in iron deficiency were more sensitive to UV. This could be explained by a distinct spectrum of specificity toward DNA lesions among the DNA repair systems, and different thresholds of induction. The response to UV damage is carried out mainly by the UvrABC system, which is part of the SOS response, but these genes (CCNA_02673, CCNA_02975, and CCNA_03076) were not induced in the DP-treated condition. In fact, these genes are poorly induced even in the absence of the *lexA* repressor (*uvrA*, 3.5-fold; *uvrC*, 1.7-fold, and *uvrB* not induced) (da Rocha et al., 2008). The DNA lesions caused by oxidative stress are distinct from the typical UV-generated DNA lesions, requiring a specific set of repair enzymes, and the concomitant exposure to oxidative stress (generated by iron limitation) and UV is probably excessive for the cell responses.

The fact that iron is a cofactor for several enzymes necessary to help protecting against ROS (e.g., Fe-containing superoxide dismutases and heme-containing catalases/peroxidases) increases the complexity of this matter in what is the cause and what is the effect. The lack of iron could be disrupting the detoxification enzymes activity to a point when the cells start accumulating ROS. On the other hand, this does not seem to be the case in the *fur* mutant, since in this case iron uptake is constitutively active and these enzymes would be functional. A possible explanation for the increase in oxidative stress in low iron medium and in the *fur* mutant is an impairment of the respiratory function, mainly due to lower synthesis of the iron–sulfur groups for enzymes of the respiratory chain. In eukaryotic cells dysfunctions that prevent the assembly of mitochondrial iron-sulfur components generate mtDNA instability and ROS, and have been treated with antioxidants (Figueira et al., 2013). It was shown that in *C. crescentus* Fur directly activates the expression of *sdhCBAD* (succinate dehydrogenase) and *nuoA-N* (NADH dehydrogenase) operons (da Silva Neto et al., 2009), and in this work several genes encoding cytochromes were downregulated in DP-treated medium and in the *fur* mutant, noteworthy the *ccoNOQP* and *cydAB* operons, which belong to the FixK regulon. Moreover, in DP-treated medium the operon encoding ATP synthase was also downregulated.

It has been reported in *E. coli* that the *fur* mutant showed a significant increase of mutagenesis in aerobic conditions and the addition of ferrozine did not reduce the frequency of mutagenesis, suggesting that this strain is suffering DNA damage (Touati et al., 1995). Furthermore, in the *E. coli* *fur* mutant the SOS response was not activated, and the mutagenesis was proposed to be due to recombination defects (Touati, 2000). It was suggested that the *fur* mutant has a permanent influx of iron, and this intracellular overload of iron could produce DNA damage that the addition of ferrozine was not able to avoid (Touati, 2000). We previously demonstrated that the *C. crescentus* *fur* null mutant is highly sensitive to exogenously added H_2_O_2_ and tert-butyl hydroperoxide (da Silva Neto et al., 2009). On the contrary to what was seen for *E. coli*, we observed that under iron limitation wild type *C. crescentus* generates oxidative stress, and the *fur* mutant is already in an endogenous oxidative state. Moreover, iron limitation (**Figure 5** and **Supplementary Figure S2**) triggered the induction of SOS system in both strains, suggesting that DNA damage did not occur by excessive iron uptake. Agreeing with this, the SOS response was not induced even when it was exposed to 100 μM of FeSO_4_ (**Supplementary Figure S2**). Taken together, these results suggest that in *C. crescentus* DNA damage is not generated by excess of free iron, although we cannot exclude the possibility that high endogenous iron levels are generating the oxidative stress observed in the *fur* mutant.

The increase in DNA damage observed in *C. crescentus* could be a consequence of the oxidative stress generated under iron limitation. Previous studies had shown that the lesions caused by H_2_O_2_ in *E. coli* are dependent on iron concentration, with higher H_2_O_2_ concentrations being necessary for the maximal induction of SOS response in low iron condition (Asad and Leitao, 1991; Asad et al., 1997). Furthermore, *E. coli* *lexA* and *recA* mutants are sensitive to H_2_O_2_ under iron limitation, suggesting that the induction of SOS response is important when the cells are treated with H_2_O_2_ in low iron condition (Asad et al., 1997). We cannot exclude the possibility that the lower levels of intracellular iron could affect not only iron metabolism, but also other ions like copper or zinc, that could lead to DNA damage. It was previously reported that high H_2_O_2_ concentration under iron limitation leads to an increase of Cu ions inside the cells causing DNA damage and the activation of SOS response (Almeida et al., 2000). However, we have to take into account that an increase in the oxidative stress was not observed in *E. coli* under iron limitation. At any case, the induction of DNA repair genes observed did not protect the cells against UV-light exposure, suggesting that either the SOS response is not at its maximum, or there is a synergistic effect of both treatments in generating DNA damage.

The *C. crescentus* *fur* mutant in M2 is under oxidative stress, but this does not lead to the induction of SOS response, since the *lexA* and *imuA* transcript levels are repressed 0.5-fold in the *fur* mutant as compared to wild type in iron sufficiency. Moreover, in the DP-treated medium the *lexA* and *imuA* transcript levels are twofold and eightfold higher in the *fur* mutant, respectively, than wt cells in the same condition. These results suggest that Fur is involved in their regulation, probably indirectly. In *Anabaena* sp 7120 FurA acts as repressor of a single-strand DNA binding protein (SSB) that plays a role in recombination and repair by protecting the single-stranded DNA, indicating a possible role of Fur in DNA repair in cyanobacteria (Kirti et al., 2017).

The results of this work showed here for the first time that iron deprivation is causing oxidative stress and DNA damage in *C. crescentus*, triggering the activation of several stress response pathways. Further experimentation will be necessary to establish whether these phenotypes are or not related, and whether this may be a common trend to other bacteria.

## Data Availability Statement

The genomic datasets analyzed for this study can be found in NCBI Assembly (ASM2200v1; GCF_000022005.1). The RNA-seq datasets generated for this study can be found in the NCBI Sequence Read Archive under accession code SRP136695.

## Author Contributions

LL performed the experiments and analyzed the data. LS conducted the RNA-seq experiments. RR, AL, and TK performed the bioinformatics analyses. NMS performed the qRT-PCR experiments. TA performed the H_2_O_2_ quantification assays. MS and MHGM performed the 8-oxo-dG quantification assays. MVM designed the experiments and analyzed the data. LL and MVM wrote the manuscript. All authors read, revised, and approved the final manuscript.

## Conflict of Interest Statement

The authors declare that the research was conducted in the absence of any commercial or financial relationships that could be construed as a potential conflict of interest.
