# Supplementary material for: Iron Deficiency Generates Oxidative Stress and Activation of the SOS Response in Caulobacter crescentus
Source: Front Microbiol. 2018 Aug 28;9:2014. doi: 10.3389/fmicb.2018.02014 (PMC6120978; doi:10.3389/fmicb.2018.02014)
Supplement: Supplementary file 1 [file Table_1.DOCX]

Supplementary Table S1: Primers used in this study

| **Target gene** | **Product** | **Primers** | **Sequences** |
| --- | --- | --- | --- |
| CCNA_03138 | peroxidase/catalase KatG | KatG 8  KatG 9 | TGGCCGATCAAGCAGAAGTA  AGGTCTTGAAGCCCATCGACT |
| CC_0088 | hypothetical protein | CC_0088 F  CC_0088 R | AGCGCGACTTTTTGGCTCAGGT  AGGTGCGCGCAAAGTCGAAGAA |
| CCNA_03876 | transcription termination factor Rho | Rho F  Rho R | GTCGAGAACGCCAACTCCAT  CGAGGGTCTTCAGGATCGC |
| CCNA_03319 | Conserved hypothetical protein ImuA | imuA F  imuA R | TCCGCCCTTCCGATCTG  CTTTGTCACCCAGCTGATCC |
| CCNA_00055 | ferric uptake regulation protein | Fur F  Fur R | GACATGAAGACCGGCAAGGTCG  CGGTGGTCGATCAGCTTGTAGC |
| CCNA_03195 | RNA polymerase sigma factor RpoH | RpoH F  RpoH R | GTTCGAGAAGCTGCAGAAGACC  ACGATCATGAGCCAAAGCCCTA |
| CCNA_03589 | RNA polymerase EcfG family sigma factor sigT | sigT F  sigT R | TCTCGAGGATCGCCTGCAAC  CGAAATCTGTAATTGCGCCGTC |
| CCNA_01979 | LexA repressor | lexA F  lexA R | CGGTGAGCACTATGTCTTGGAA  GATGATCACGTAGTCGCCATCG |
