# Supplementary material for: Iron Deficiency Generates Oxidative Stress and Activation of the SOS Response in Caulobacter crescentus
Source: Front Microbiol. 2018 Aug 28;9:2014. doi: 10.3389/fmicb.2018.02014 (PMC6120978; doi:10.3389/fmicb.2018.02014)
Supplement: Supplementary file 3 [file Data_Sheet_1.pdf]

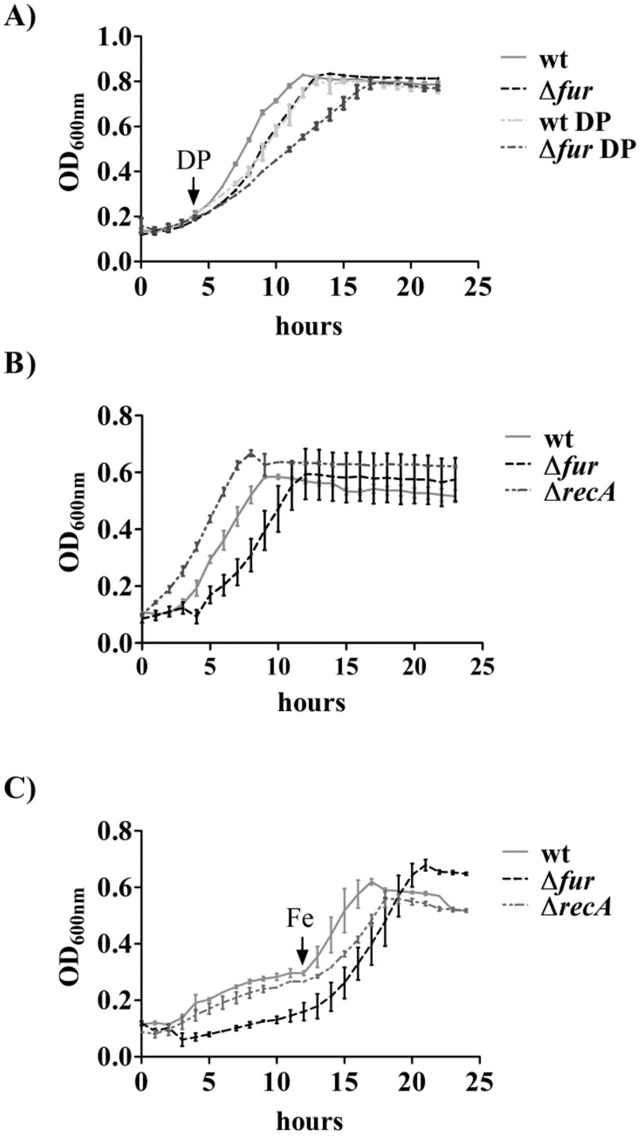

Supplementary Figure S1. Growth of *C. crescentus* strains in media with different iron availability. (A) Duplicate cultures of the NA1000 and *fur* mutant strains were incubated in M2 for 4 h, and one of the cultures from each strain received 100 µM DP (arrow). (B) Cultures of NA1000 (wt), *fur* and *recA* mutants grown in M2 medium were centrifuged and resuspended in the same volume of M2. (C) Cultures of NA1000 (wt), *fur* and *recA* mutants grown in M2 medium were centrifuged and resuspended in the same volume of M2 medium without added iron at time 0h. After incubation for 12 h, 10 µM $FeSO_4$ was added to the cultures (arrow).
