# Supplementary material for: Iron Deficiency Generates Oxidative Stress and Activation of the SOS Response in Caulobacter crescentus
Source: Front Microbiol. 2018 Aug 28;9:2014. doi: 10.3389/fmicb.2018.02014 (PMC6120978; doi:10.3389/fmicb.2018.02014)
Supplement: Supplementary file 4 [file Data_Sheet_2.pdf]

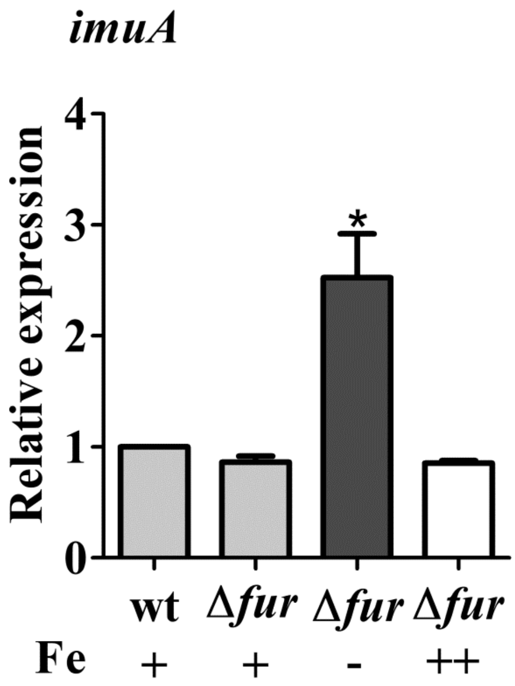

Supplementary Figure S2. Expression of *imuA* in the *fur* mutant. Gene expression was assessed by RT-qPCR from wild type NA1000 (wt) and the Fur mutant (*Δfur*) cultures grown in either M2, iron limited M2 (no iron added) for 4 h and iron-suplemented M2 (with 100 mM $FeSO_4$) for 2 h. Bars with asterisks (*) are significantly different from wt in M2 medium; (**) indicates that the results of *Δfur* in M2 and *Δfur* in iron-suplemented M2 are significantly different from each other ($P < 0.05$) by One Way ANOVA test.
